# Lipid Mediated Regulation of Adult Stem Cell Behavior

**DOI:** 10.3389/fcell.2020.00115

**Published:** 2020-02-28

**Authors:** Marie Clémot, Rafael Sênos Demarco, D. Leanne Jones

**Affiliations:** ^1^Department of Molecular, Cell and Developmental Biology, University of California, Los Angeles, Los Angeles, CA, United States; ^2^Eli and Edythe Broad Center of Regenerative Medicine and Stem Cell Research, University of California, Los Angeles, Los Angeles, CA, United States; ^3^Molecular Biology Institute, University of California, Los Angeles, Los Angeles, CA, United States

**Keywords:** lipid, metabolism, fatty acids, stem cells, niche

## Abstract

Adult stem cells constitute an important reservoir of self-renewing progenitor cells and are crucial for maintaining tissue and organ homeostasis. The capacity of stem cells to self-renew or differentiate can be attributed to distinct metabolic states, and it is now becoming apparent that metabolism plays instructive roles in stem cell fate decisions. Lipids are an extremely vast class of biomolecules, with essential roles in energy homeostasis, membrane structure and signaling. Imbalances in lipid homeostasis can result in lipotoxicity, cell death and diseases, such as cardiovascular disease, insulin resistance and diabetes, autoimmune disorders and cancer. Therefore, understanding how lipid metabolism affects stem cell behavior offers promising perspectives for the development of novel approaches to control stem cell behavior either *in vitro* or in patients, by modulating lipid metabolic pathways pharmacologically or through diet. In this review, we will first address how recent progress in lipidomics has created new opportunities to uncover stem-cell specific lipidomes. In addition, genetic and/or pharmacological modulation of lipid metabolism have shown the involvement of specific pathways, such as fatty acid oxidation (FAO), in regulating adult stem cell behavior. We will describe and compare findings obtained in multiple stem cell models in order to provide an assessment on whether unique lipid metabolic pathways may commonly regulate stem cell behavior. We will then review characterized and potential molecular mechanisms through which lipids can affect stem cell-specific properties, including self-renewal, differentiation potential or interaction with the niche. Finally, we aim to summarize the current knowledge of how alterations in lipid homeostasis that occur as a consequence of changes in diet, aging or disease can impact stem cells and, consequently, tissue homeostasis and repair.

## Introduction

Adult stem cells, also referred to as “tissue” stem cells, are relatively undifferentiated cells that have the ability to self-renew and produce differentiating progeny. Consequently, they are crucial for maintaining tissues that must be constantly replaced and also serve as a reservoir of cells that can be used to repair damaged tissues after wounding or exposure to environmental insults. Stem cell behavior is influenced by the integration of cell-intrinsic factors, extrinsic cues provided by the local microenvironment (or “niche”) and systemic factors (Voog and Jones, 2010). Furthermore, the capacity of stem cells to self-renew or differentiate can be attributed to distinct metabolic states and it is now becoming apparent that metabolism plays instructive roles in stem cell fate decisions (Ito and Suda, 2014; Chandel et al., 2016; Folmes and Terzic, 2016; Mana et al., 2017; Shyh-Chang and Ng, 2017).

Lipids can be broadly defined as any organic compound insoluble in water but soluble in organic solvent. Therefore, lipids constitute an extremely vast class of biomolecules, which is reflected in the high diversity of roles they assume in a cell (Fahy et al., 2011) (Table 1). Glycerophospholipids, in particular phosphatidylcholine (PC), phosphatidylethanolamine (PE), phosphatidylserine (PS), and phosphatidylinositol (PI), as well as sphingolipids and cholesterol, primarily serve as building blocks for membranes and organelles (van Meer and de Kroon, 2011). Fatty acids (FAs) provide a source of energy, as they can be stored in the form of triglycerides (TAG) in lipid droplets (LDs). When FAs are needed, TAG undergoes lipolysis, and the resulting FAs can be broken down through β-oxidation in mitochondria or in peroxisomes in the case of very long chain FAs, to produce energy under the form of ATP and reducing power (Houten and Wanders, 2010). In addition, lipids can act as signaling molecules through specific lipid-protein interactions. Signaling lipids, acting as extracellular ligands or intracellular second messengers, participate in the regulation of various biological processes, including cell proliferation, cell death, cell migration, gene expression or immune reactions such as inflammation. An increased appreciation of the involvement of lipids in metabolic diseases such as obesity, atherosclerosis, stroke, hypertension, diabetes and cancers (Wymann and Schneiter, 2008), together with technological advances in mass spectrometry and computational methods and global efforts like the LIPID MAPS (Metabolites And Pathways Strategy) consortium (Fahy et al., 2005), have pushed lipid biology to the forefront of metabolism research.

**TABLE 1:** Classes of lipids.

**Categories**	**Composition**	**Function**	**Classes or examples**
Fatty acids	Carboxylic acid + hydrocarbon chain; synthesized by chain elongation of an acetyl-CoA with malonyl-CoA	Building blocks to complex lipids	SFA, MUFA, PUFA
Glycerolipids	FA + glycerol; may have sugar residues	Energy storage, cell signaling	TAG, DAG, MAG
Glycerophospholipids	Polar head group + glycerol group, may contain LCFA	Membrane composition, cell signaling	PC, PE, PS, PI, PA
Sphingolipids	Sphingoid base + LCFA-CoA	Membrane and lipoprotein composition, cell signaling	Ceramides, Phosphosphingolipids, glycosphingolipids
Sterols	Hydroxyl group + steroid	Membrane, precursor to hormones and vitamins	Cholesterol, bile acids
Prenols	Isopentenyl diphosphate + dimethylallyl diphosphate	Antioxidants, vitamin precursor	Quinone, ubiquinone, Vitamin E, Vitamin K
Saccharolipids	FAs + sugar backbone	Membrane components	Glucosamine
Polyketides	Acetyl + propionyl polymerization	Secondary metabolites	Tetracycline, erythromycin

*List of the different categories of lipids, as defined by the LIPID MAPS consortium, with summaries of their average composition, functions and classes. FA, fatty acid; SFA, short chain fatty acid; MUFA, mono-unsaturated fatty acid; PUFA, poly-unsaturated fatty acid; TAG, triacylglycerol; DAG, diacylglycerol; MAG, monoacylglycerol; PC, phospatidylcholine; PE, phosphatidylethanolamine; PS, phosphatidylserine; PI, phosphatiylinositol; PA, phosphatidic acid; LCFA-CoA, long-chain fatty acid-coenzyme A.*

In this review, we intend to summarize growing evidence implicating lipids in the regulation of stem cell behavior. A better understanding of the mechanisms by which lipids act will contribute to improve the appreciation of how imbalances in lipid homeostasis can cause or contribute to alterations in tissue homeostasis and regeneration. In addition, a better appreciation for the roles that lipids play in stem cells will facilitate the development of novel approaches to enhance stem cell expansion and differentiation *in vitro* for use in regenerative medicine. Taken together, this knowledge may ultimately allow for the control of stem cell behavior in patients, by modulating lipid metabolic pathways pharmacologically or through diet.

## Lipidomics and Lipids Enriched in Stem Cells

The lipidome is the complete set of lipids present within a cell, a tissue or an organism. It is a subset of the metabolome, which also includes the three other major classes of biological molecules: amino acids, sugars and nucleic acids (Fahy et al., 2011). It has become clear that the lipidome, similar to the transcriptome and the proteome, is dynamic and can be actively remodeled upon different physiological conditions, diets and stimuli (García-Cañaveras et al., 2017; Lydic and Goo, 2018). Thus, improved approaches for lipidomics have contributed significantly to the development of diagnostic tools and therapeutic strategies for metabolic diseases (Lydic and Goo, 2018).

### Lipidomics

**FIGURE 1:**
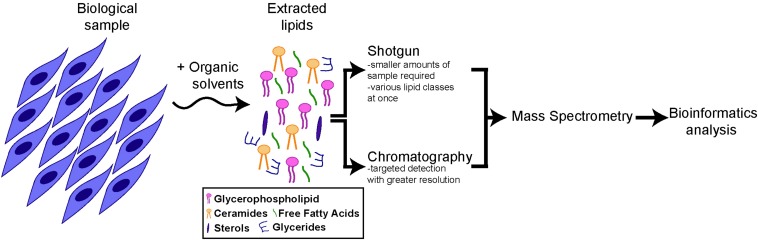
Schematics of lipidomics analysis. All main lipids categories can be extracted from cells or tissue samples through organic solvents. After extraction the lipid composition of the samples can be analyzed directly (“shotgun” approach) or after chromatography, by mass spectrometry and bioinformatics analysis (for more details, see section “Lipidomics and Lipids Enriched in Stem Cells”).

Approaches to provide global profiles of lipid species, referred to as lipidomics, recently experienced significant advances, due to the advent of next-generation mass spectrometry (MS) instruments in combination with bioinformatics (Wenk, 2005, 2010; German et al., 2007; Shevchenko and Simons, 2010). Lipidomics involves multiple steps (Lydic and Goo, 2018) (Figure 1). First, lipids are extracted from the biological sample using organic solvents. Lipids can then be ionized and directly infused into a mass spectrometer (as in the case of “shotgun” lipidomics) or separated by chromatography, prior to detection by MS. Both methods are complementary, as the shotgun method allows lipid profiling from a smaller amount of biological sample and the simultaneous analysis of various classes of lipids, while chromatography/MS enables a more targeted analysis with the detection of structurally close lipids within a single class. Finally, identified lipids are quantified, using a ratio against internal standard(s). In the case of targeted lipidomics, labeled lipids can be included for absolute quantification. For shotgun lipidomics, exogenous lipids representative of the main lipid classes of interest are generally used, with lipid cocktails being commercially available for this purpose.

### Lipidomics in Stem Cells

#### Pluripotent Stem Cells

In 2010, Yanes and colleagues were one of the first to provide a characterization of stem cells with an untargeted metabolomics approach. When comparing the metabolomes of mouse embryonic stem cells (mESCs) and differentiated neurons and cardiomyocytes, lipid messengers and inflammatory mediators, such as arachidonic acid, linolenic acid, diacylglycerols, glycerophosphocholines, glycerophosphoglycerols, and eicosanoids, were among the most upregulated metabolites in mESCs, relative to differentiated cells. In addition, the degree of unsaturation was significantly higher in mESCs compared to differentiated cells. Differentiated cells showed increased levels of saturated free FAs and acyl-carnitines, which consist of fatty acyl–CoA conjugated to carnitine and are intermediates for the transport of FAs into the mitochondria for β-oxidation. Because carbon-carbon double bonds are highly reactive under oxidative conditions, the authors propose that the high degree of unsaturation observed in mESCs could allow for the maintenance of “chemical plasticity.” As oxidative pathways, such as the eicosanoid signaling pathway for which substrates were found to be enriched in mESCs, promote differentiation, control of the reduction-oxidation (redox) status of mESCs could be a mechanism to regulate stem cell fate (Yanes et al., 2010). Accordingly, inhibition of the eicosanoid pathway promoted pluripotency and the maintenance of high levels of unsaturated FAs, while metabolites that promote fatty acid oxidation (FAO), such as acyl-carnitine, enhanced differentiation (Yanes et al., 2010). Importantly, human ESCs also appeared to be enriched in metabolites with a high degree of unsaturation and preliminary data suggested that human ESC differentiation involves oxidative reactions as for mESCs (Yanes et al., 2010). Of note, an untargeted metabolomics approach was also applied to induced pluripotent stem cells (iPSCs) and the metabolomics profile of iPSCs shared much more similarities with ESCs than with the cell of origin, which may be referred to as a pluripotent metabolomics signature (Panopoulos et al., 2012). Interestingly, however, the levels of unsaturated FAs were lower in iPSCs than in ESCs.

In 2010 as well, Park and colleagues performed a more targeted lipidomics analysis of mESCs focused on changes in sphingolipids and ceramides (Cer) species during the differentiation to embryoid bodies (EBs), a model that recapitulates the early stages of embryonic development (Park H. et al., 2010). They found that C16-Cer, a lipid species involved in apoptosis, generally decreased during transition from ESCs to EBs. A higher level of apoptosis in ESCs was suggested to participate in the elimination of residual pluripotent cells. In addition, there was an increase in very long chain dihydroceramides (DHCer), which was proposed to play a role in developmental autophagy taking place during the formation of the inner cell mass. In parallel, changes in the expression of genes encoding enzymes involved in the biosynthesis of sphingolipids and ceramides were assessed and appear to confirm the lipidomics data.

Another study reported the changes of the lipid profile of stem cells during differentiation of iPSCs into hepatocyte-like cells (Kiamehr et al., 2017). During hepatic differentiation and maturation, an overall increase in the FA chain length of sphingolipids was observed. Among phospholipids, polyunsaturated fatty acid (PUFA)-containing lipid species increased in the more mature stages. Importantly, the amount of FAs and lipids available in the different culture media successively used to induce differentiation and maturation of the iPSCs into hepatocyte-like cells was greatly reflected in the lipid composition of the cells at different stages. This was also observed in mesenchymal stem cells (MSCs; see below) (Chatgilialoglu et al., 2017) and constitutes a possible caveat when analyzing the lipidome of isolated stem cells maintained *ex vivo*.

#### Mesenchymal Stem Cells

Several studies analyzed the lipidome of MSCs (Kilpinen et al., 2013; Campos et al., 2016; Chatgilialoglu et al., 2017; Lu X. et al., 2019). Specifically, the glycerophospholipid profiles of human bone MSCs from young and old donors and across passages during *in vitro* culture were assessed (Kilpinen et al., 2013; Chatgilialoglu et al., 2017; Lu X. et al., 2019). Small changes in membrane glycerophospholipids can have important consequences in terms of signaling mediated by lipid derivatives. Hence, it is particularly relevant to determine changes induced by *in vitro* culture conditions, especially if the stem cells were to be used for therapy.

During long-term culture, total PI and total lysoPC consistently increased, with a more pronounced effect when MSCs were isolated from young donors (Kilpinen et al., 2013). Freshly isolated MSCs had high omega-6 FA content, which decreased in culture (Chatgilialoglu et al., 2017). In addition, the proportion of individual saturated FAs increased in late passages, whereas individual mono-unsaturated FAs (Kilpinen et al., 2013) and poly-unsaturated FAs (Chatgilialoglu et al., 2017) decreased. Based on these findings, a tailored culture medium that minimizes changes in the membrane FA composition of MSCs across passages was proposed (Chatgilialoglu et al., 2017).

Recently, Lu et al. also characterized alterations of the lipidome during passaging of MSCs, as a way to uncover changes that may play an important role in the aberrant differentiation of MSCs during aging (Lu X. et al., 2019). Aging is one condition that biases MSC differentiation toward adipocyte fate at the expense of osteoblasts, which may contribute to age-related loss of bone mass and osteoporosis. This study used ultraperformance liquid chromatography coupled to mass spectrometry (UPLC-MS), as this lipidomics approach is more sensitive than the shotgun-based analysis and able to effectively cover lipid species with low abundance. The results obtained were largely consistent with previous studies described above (Kilpinen et al., 2013), as lysoPC and saturated FAs were similarly found to increase with passaging (Lu X. et al., 2019). In contrast, whereas PI species exhibited a significant increase with passaging in the previous study (Kilpinen et al., 2013), in this study a decrease was observed. The reduction of PIs observed by Lu et al. is somewhat inconsistent with the transcriptomics analysis conducted in parallel that showed an increase in expression of the enzymes involved in the conversion of phosphatidic acid to PIs, suggesting that PI biosynthesis is likely more active.

Finally, to better understand the mechanisms underlying MSCs anti-inflammatory properties, a primary reason for the use of MSCs in therapy, Campos et al. (2016) examined the variations of the MSC lipidome under pro-inflammatory conditions. Overall, pro-inflammatory stimuli caused no significant differences in phospholipid class levels, but variations were found in specific molecular species within all classes, except PI. These variations can be correlated with MSCs immunomodulatory properties. The lipidome of the untreated MSCs was consistent with previous results (Kilpinen et al., 2013), with the exception of the presence of sphingomyelins (Campos et al., 2016), which had not been previously identified.

#### Adult Stem Cells

Despite improvements in lipidomics, the number of studies that have analyzed specifically the lipidome of adult stem cells remains rather limited, especially in comparison to the numerous studies investigating the transcriptome and the proteome. In 2007, what appears to be the first lipidomics analysis in stem cells focused on glycerophospholipids in adult mouse retinal stem cells (Li et al., 2007). Interestingly, among PE, PS and PI, proliferating retinal stem cells showed a significant enrichment for saturated FAs and a decrease in very long chain PUFAs, when compared to differentiated retinal cells. The changes correlated with lower membrane fluidity in retinal stem cells. The same year, another study performed lipidomics on hematopoietic progenitor cells undergoing apoptosis in response to growth factor deprivation (Fuchs et al., 2007). Specifically, this study characterized the extent of changes occurring in membrane glycerophospholipids during apoptosis and found a decrease in diacyl-PC and diacyl-PE species, with a concomitant increase in ether-linked glycerophosphocholines and -ethanolamines in the membranes of the hematopoietic progenitors.

Imaging MS can be applied to tissue sections to characterize the lipidome *in situ* and obtain information on the local distribution of lipid species. This approach was used to characterize the lipidome of the human subventricular zone (SVZ), an important center for neurogenesis in the brain, and showed variations in lipid composition among the four layers of the SVZ (Hunter et al., 2018). However, they were not able to identify lipids specific to the layer that contains neural stem cells (NSCs), possibly due to the heterogeneity of cell types within this region and a greater diversity of lipids. Yet, this may provide a basis for further, more refined, lipidomics analysis in neural stem and progenitor cells.

### Lipids That Are Enriched in Stem Cells

Lipidomics in stem cells potentially offers the possibility to identify specific lipid signatures that could be used as biomarkers to identify and sort stem cells. In addition, changes in the lipidome throughout differentiation of stem cells could reflect changes in substrate availability and could shed light on the mechanisms underlying the differentiation process (Pébay and Wong, 2013). Lipidomics analyses should be performed quickly upon isolation of the stem cells, whenever possible, to provide a snapshot of the lipid profile *in vivo*, prior to culture and expansion. Indeed, current lipidomics data suggest that the composition of the media should be taken into account when analyzing the lipid profile of stem cells maintained in culture. For example, a recent study found that maintaining hPSCs in a lipid-deprived culture medium was responsible for the activation of a lipid biogenesis program in hPSCs that maintained these cells in a more naïve pluripotent state (Cornacchia et al., 2019). Consequently, the authors propose that manipulating lipid availability could be used to modulate the pluripotent state.

More lipidomics analyses are required, particularly in adult stem cells, to establish the extent of the similarities between different types of stem cells and whether this could allow to identify lipid species that would regulate “universal” stem-cell properties, such as self-renewal or maintenance in a multipotent state. Remarkably, the existing data described here suggest that different types of stem cells, including ESCs, iPSCs and MSCs, show an enrichment for lipids containing unsaturated FAs, although this may not be true for retinal stem cells that have less poly-unsaturated FAs than their differentiated counterparts. It would be interesting to determine in this model whether the overall unsaturation level is higher in stem cells. Nevertheless, if this trend is confirmed in other types of adult stem cells, this could corroborate the model proposed by Yanes et al. (2010), according to which the degree of unsaturation would be correlated with a “chemical plasticity” required to maintain stem cells in a pluri- or multipotent state.

## Lipid Metabolism in Stem Cells

### Fatty Acid Oxidation

**FIGURE 2:**
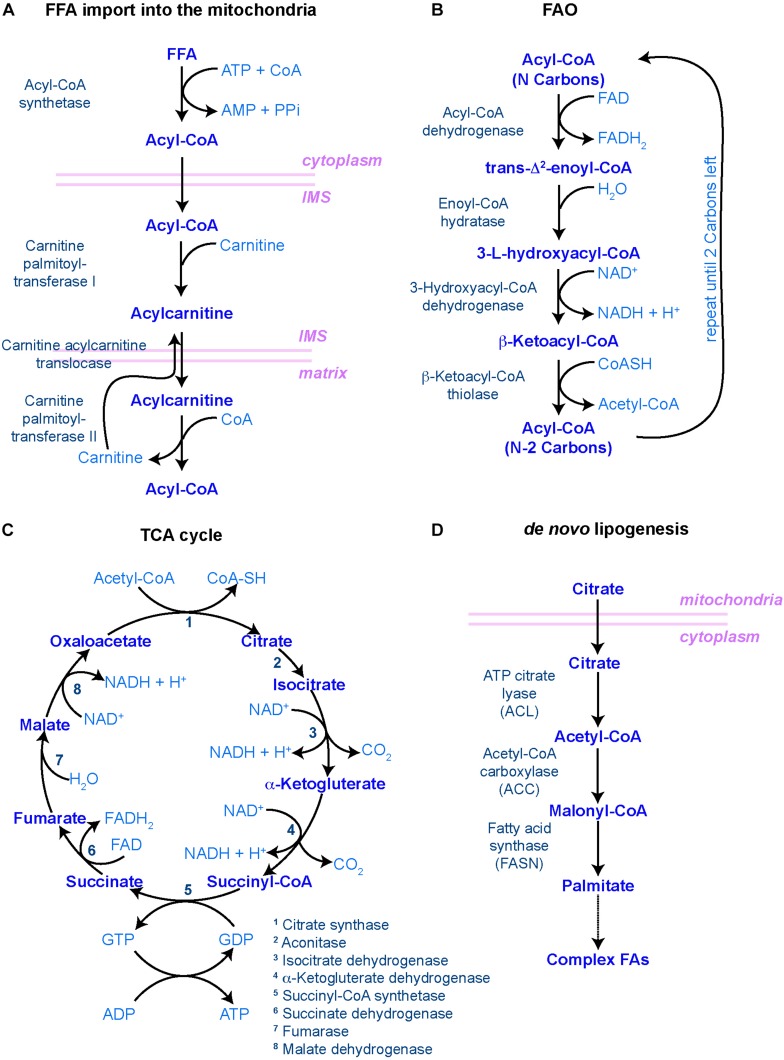
Main biochemical pathways involved in lipid metabolism. **(A–C)** Pathways involved in lipid catabolism. **(A)** In order to be catabolized, free fatty acids (FFAs) must first travel to the mitochondrial matrix through the carnitine shuttle. IMS – inner mitochondrial space. **(B)** Once incorporated into the matrix, fatty acyl-CoA molecules undergo rounds of FAO, resulting in one molecule of acetyl-CoA (with two carbon atoms) and another molecule of fatty acyl-CoA with the original number of carbon atoms minus 2. This process is repeated until only two carbon atoms are left. **(C)** The resulting acetyl-CoA molecules are then incorporated into the tricarboxylic acid (TCA) cycle, where the carbon atoms will be fully reduced into CO_2_, generating NADH, FADH2, GTP (and hence, ATP) and H^+^ protons to fuel the oxidative phosphorylation process in the mitochondrial cristae, regulated by the electron transport chain. **(D)** In order to make new FAs (i.e. lipid anabolism), citrate leaves the mitochondria to start the *de novo* lipogenesis pathway. In all diagrams, enzymes are in dark blue to the left [in panel **(C)**, they are numbered]; main metabolites are in the middle in blue; and co-factors/resulting products are in light blue to the right. Mitochondrial membranes are represented in pink.

When FAs are taken up for catabolism, they can be transported into peroxisomes, in case of very long chain FAs, or the mitochondria, where a step-wise series of redox reactions, collectively called FAO or β-oxidation, breaks down FAs into acetyl-CoA (Figure 2). This process is tightly regulated by a set of transporters and enzymes that facilitate the entry of FAs into the mitochondria. The rate-limiting step of this process is controlled by carnitine palmitoyltransferase I (CPT1), a mitochondrial enzyme that catalyzes the transfer of acyl groups from fatty acyl-CoA molecules to L-Carnitine for further entry into the mitochondrial matrix, where oxidation of the fatty acyl groups happens (Figure 2). When inside the mitochondrial matrix, acetyl-CoA enters the tricarboxylic acid (TCA) cycle and generate NADH and FADH_2_, which in turn are oxidized in the electron transport chain (ETC) to fuel oxidative phosphorylation (Figure 2) (Houten and Wanders, 2010).

In vertebrates, the peroxisome proliferator-activated receptor (PPAR) family of transcription factors acts as major regulators of FA metabolism. PPARs bind to co-factors, usually lipids, and translocate into the nucleus, where they dimerize with the retinoid X receptor (RXR) to bind to peroxisome proliferator hormone response elements (PPREs). These sequences are most commonly found in the promoter regions of genes involved in lipid metabolism. There are three major classes of PPARs, with PPARα and PPARβ/δ being associated with genes involved in lipid catabolism, while PPARγ is associated with genes involved in lipid anabolism. PPAR families and splice variants are differentially expressed in a tissue specific manner (Berger and Moller, 2002; Pawlak et al., 2015).

Recently, a model has emerged to suggest that FAO plays a crucial role in the maintenance of several adult stem cell populations. Although the precise mechanisms through which FAO acts to maintain stem cells may differ depending on the nature and behavior of the stem cell population (e.g. quiescent versus highly proliferative stem cells), data have shown that adult stem cells are negatively affected by pharmacological or genetic ablation of components of the FAO machinery.

Hematopoietic stem cells (HSCs) have been shown to rely on glycolysis for energy homeostasis (Gan et al., 2010; Gurumurthy et al., 2010; Nakada et al., 2010; Simsek et al., 2010; Takubo et al., 2010). However, in 2012, Ito and colleagues made the surprising discovery that HSCs rely on FAO for asymmetric division and, thus, maintenance. Indeed, HSCs displayed higher rates of FAO than differentiated progeny, and FAO inhibition by the CPT1 inhibitor etomoxir in HSCs led to a significant reduction in long-term HSC occupancy in bone marrow of irradiated mice upon transplantation. Inhibition of FAO *in vitro* triggered loss of quiescence in HSCs combined with excessive commitment, resulting in HSC exhaustion and inability to sustain the hematopoietic compartment (Ito et al., 2012). Moreover, loss of PPARδ in HSCs led to decreased ATP levels and exit from quiescence, while activation of PPARδ resulted in an increase in ATP levels and enhanced HSC function, as measured by colony formation and differentiation potential (Ito et al., 2012). Using a combination of PPARδ agonists and etomoxir, FAO was shown to act downstream of PPARδ in the regulation of HSC maintenance. The same group further detailed the cellular mechanisms controlling HSC maintenance by showing that Pink1/PARKIN-mediated mitophagy was also important for controlling FAO rates and the maintenance of HSCs (Ito et al., 2016).

Adult neural stem/progenitor cells (NSPCs) are also quiescent and rely on FAO for their maintenance (Knobloch et al., 2017). Similar to what was observed for HSCs, quiescent NSPCs express higher levels of CPT1A and have higher rates of FAO than proliferating NSPCs (Stoll et al., 2015; Knobloch et al., 2017). Strikingly, the presence of malonyl-CoA, which inhibits the rate-limiting step in FAO, was sufficient to induce exit from quiescence to proliferation (Knobloch et al., 2017), indicating that the tight regulation of FAO plays an important role in the regulation of NSPC behavior. FAO is also required in embryonic NSCs, where CPT1A and the breakdown of FAs from LDs have been shown to regulate the asymmetric divisions and maintenance of these stem cells (Xie et al., 2016).

Male germline stem cells (GSCs) in *Drosophila melanogaster* have been recently characterized to also rely on the mitochondrial uptake of lipids to prevent the switch to lipid anabolism and consequent loss of stem cell identity (Sênos Demarco et al., 2019). Similar to the proposed mechanism of mitochondrial control of FAO utilization seen in HSCs (Ito et al., 2016), active mitochondria are required to promote lipid catabolism and GSC maintenance. Disruption of mitochondrial activity in these cells, via blocking mitochondrial fusion, led to the accumulation of LDs, activation of Target of Rapamycin (TOR), and precocious germ cell differentiation (Sênos Demarco et al., 2019).

Other stem cell populations, such as intestinal stem cells (ISCs) and skeletal muscle stem cells (also known as satellite cells), also rely on FAO for maintenance. In *Drosophila*, inhibition of lipolysis or FAO led to ISC necrosis (Singh et al., 2016). In mammals, both a high fat diet (HFD) and fasting regimens have been shown to promote FAO through PPARδ, and CPT1A in the case of fasting. Both led to an increase in ISC number and function (Beyaz et al., 2016; Mihaylova et al., 2018). However, while fasting improved ISC function during aging and regeneration (Mihaylova et al., 2018), HFD enhanced the capacity of ISCs to promote tumors upon loss of *Apc* (Beyaz et al., 2016). A recent study confirmed that FAO is required for ISC maintenance under normal conditions in mice (Chen et al., 2019). This study showed that cells in the intestinal crypts, including ISCs, displayed a higher capacity to import FAs than differentiated cells in villi. In addition, ISCs express high levels of hepatocyte nuclear factor 4 (HNF4) transcription factors. These transcription factors activate the transcription of FAO genes, which are required for the maintenance of ISCs (Chen et al., 2019). ISC loss was rescued by supplementation of acetate, suggesting that the essential role of HNF4 factors for ISC maintenance involves their role in promoting FAO. Therefore, in ISCs, high levels of FAO are regulated, at least in part, at the transcriptional level.

In muscle, quiescent satellite cells undergo a transition from FAO to glycolysis during activation and proliferation, which mediates a decrease in NAD^+^ levels and consequent decreased SIRT1 activity. Reduced SIRT1 activity resulted in changes in the epigenome, particularly elevated H4K16 acetylation, and the expression of differentiation genes (Ryall et al., 2015).

The promotion and maintenance of cells that seed and maintain tumors, known as cancer stem cells (CSCs), also rely on FAO, a process that could be exploited as anti-tumor therapy. As mentioned above, high FAO rates are associated with the development of intestinal tumors (Beyaz et al., 2016; Singh et al., 2016). Additionally, FAO also supports CSC maintenance in the blood, liver and breast (Samudio et al., 2010; Chen et al., 2016; Wang T. et al., 2018).

In sum, in contrast to initial assumptions that stem cells would rely disproportionately on glycolysis, FAO seems to be an essential factor in the maintenance of many adult stem cells. Inhibition of FAO often results in a reduction in stem cell number, although the precise mechanism(s) by which stem cells are lost may differ; changes in asymmetric division, proliferation rates, gene expression, and cell death have all been implicated in a decrease in stem cell number. It remains to be seen whether a requirement for specific FAO-derived metabolites will be similar or different among adult stem cell populations.

### *De novo* Lipid Synthesis in Stem Cells

*De novo* lipogenesis is the formation of FAs from acetyl-CoA, malonyl-CoA and NADPH. This process takes place in the cytoplasm and involves two key enzymes: acetyl-CoA carboxylase (ACC) and fatty-acid synthase (FASN) (Figure 2D). The activity of ACC is rate-limiting and, as such, highly regulated both at the level of ACC expression, through transcription factors such as Sterol Regulatory Elements Binding Protein 1 (SREBP-1) and PPAR-γ, and at the level of its activation through multiple signaling pathways involving for instance AMP-activated kinase (AMPK) or thyroid hormones (Brownsey et al., 2006). ACC catalyzes the production of malonyl-CoA, an essential substrate for FASN and FA synthesis and a potent inhibitor of CPT1, the rate-limiting enzyme of mitochondrial FAO. Because of this dual function, the availability of malonyl-CoA, downstream of ACC, has been proposed to act as a rheostat by regulating the balance between anabolic lipogenesis and catabolic FAO (Folmes et al., 2013). *De novo* synthesized FAs can then serve as substrates for production of membrane lipids, be stored in the form of TAG to later generate energy through β-oxidation, or provide metabolites implicated in protein modification and signaling networks. Therefore, lipid anabolism is likely to contribute to stem cell function via multiple mechanisms. Yet, only a few studies investigated its impact on adult stem cells directly.

In a key study, *de novo* lipogenesis, and in particular FASN activity, was shown to be specifically elevated in mouse NSPCs, relative to differentiated neuronal cells (Knobloch et al., 2013). Lipogenesis was required for stem cell proliferation and, therefore, for normal neurogenesis to proceed. On the other hand, Spot14 (also called thyroid hormone responsive protein, THRSP) was identified as a repressor of NSPC proliferation. Mechanistically, Spot14 decreases FASN activity by dimerizing with Mig12, an activator of ACC, thereby inhibiting its function and causing a decrease of ACC-mediated malonyl-CoA production, leading in a reduction in FA synthesis (Knobloch et al., 2013). Of note, it was suggested that cholesterol biosynthesis, which occurs through an independent pathway, is also required for NSPC self-renewal and maintenance in the developing mouse forebrain, as NSPCs with mutations in enzymes involved in this pathway exhibit premature differentiation into neurons, causing exhaustion of the stem cell pool (Driver et al., 2016). Importantly, as in the case of NSPCs, increased lipid anabolism in *Drosophila* GSCs through the activation of SREBP also resulted in stem cell loss (Sênos Demarco et al., 2019), suggesting that a conserved mechanism may be at play across stem cell populations.

Fatty acids produced by FASN can then be elongated and desaturated, through the action of various enzymes, and participate in the formation of more complex lipids, such as membrane glycerophospholipids. Diverse forms of membrane lipids can be produced by adding different degrees of unsaturation. Lysophosphatidylcholine acyltransferase 3 (Lpcat3) is one enzyme participating in such reactions, catalyzing the incorporation of poly-unsaturated FAs on lysophospholipids. Remarkably, specific loss of function of Lpcat3 in mouse intestinal crypts led to hyperproliferation of ISCs and progenitors without affecting differentiated enterocytes, both *in vivo* and in organoids (Wang T. et al., 2018). Lipidomics on isolated Lpcat3 deficient crypts showed a selective decrease of polyunsaturated PC and increase in saturated and monounsaturated PC, which could affect membrane fluidity. Loss of Lpcat3 induced a strong transcriptional upregulation of sterol biosynthesis enzymes, driven by an increase of SREBP-2 activity and, accordingly, an increase of cholesterol in the crypts. Inhibition of cholesterol synthesis in Lpcat3-deficient ISCs rescued the hyperproliferation phenotype, while increasing cholesterol was sufficient to induce ISC proliferation (Wang B. et al., 2018).

Interestingly, *de novo* FA synthesis is also essential for pluripotency in mESCs and during reprogramming (Wang et al., 2017): ACC and FASN are upregulated during reprogramming to pluripotent cells and lipogenesis is enhanced, while lipogenesis decreased during induction of differentiation. Likewise, single human pluripotent stem cells (hPSCs) require lipid synthesis for their survival, although treatment with lipid synthesis inhibitors does not affect already established hPSC colonies (Romani et al., 2019).

Taken together, current data suggest that while FAO seems to be required for the maintenance of adult stem cells, *de novo* lipogenesis appears required and sufficient to promote stem cell activity and differentiation. One possibility is that *de novo* lipogenesis and lipid incorporation enhance the ability of stem cells to rapidly expand the plasma and organellar membranes, which is crucial for cell division and often associated with membrane remodeling during differentiation. Another possibility is that lipid biosynthesis may generate signaling lipids playing a role in the differentiation program. Moving forward, lipidomics analyses in purified stem cells will help revealing whether energy and redox (in the forms of ATP and NADH/NAD^+^) or specific lipid metabolites play a role in the maintenance of adult stem cell populations. Metabolomics analyses should also provide insights into the utilization of *de novo* generated lipids in response to proliferation and differentiation cues.

## Mechanistic Insights Into How Lipids Regulate Stem Cell Behavior

**FIGURE 3:**
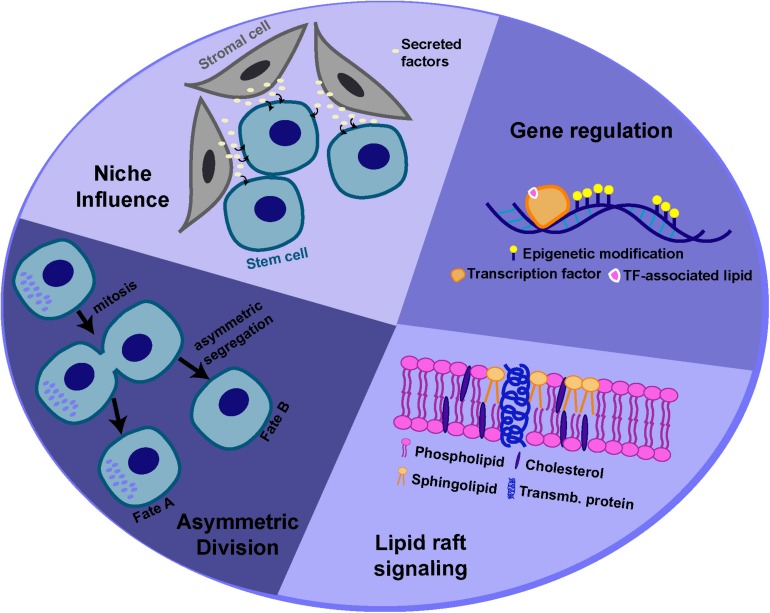
Potential mechanisms for lipid-mediated regulation of stem cell behavior. Lipids and lipid metabolism may regulate stem cell behavior through different mechanisms, including modulation of asymmetric cell division, interactions with the niche, cell signaling and gene expression.

Adult stem cells are capable of dividing in such a way that the daughter cells produced can adopt different fates. This “asymmetry,” with respect to cell fate, can be regulated by both intrinsic factors and extrinsic cues, often provided by the surrounding microenvironment, known as the “niche” (Watt and Hogan, 2000; Morrison and Kimble, 2006; Knoblich, 2008). However, stem cells must also be able to divide symmetrically to produce two stem cell daughters or daughter cells destined to differentiate (i.e. for tissue repair). The ability of stem cells to maintain tissue homeostasis by switching from asymmetric to symmetric outcomes, as the tissue demands, is achieved by “populational asymmetry.” In this section, we will describe some of the potential mechanisms by which lipid metabolism or specific lipid species can influence either self-renewal or maintenance of a multipotent state, through regulating asymmetric stem cell divisions and/or interaction with the niche or through regulating signaling and gene expression (Figure 3).

### Lipids and Asymmetric Cell Division

The establishment of cellular polarity is one mechanism by which an asymmetric outcome can be achieved, and cell polarity often depends on specific membrane phospholipids and more specifically phoshoinositides (PIs). Although not a stem cell *per se*, the early *Caenorhabditis elegans* embryo has served as an excellent model to uncover mechanisms of asymmetric division (reviewed in Schneider and Bowerman, 2003), and several studies have shown the involvement of lipids in generating polarity in this model. First, depletion of FAs or loss of ACC activity in the *C. elegans* one-cell embryo caused mislocalization of the PAR-3/PAR-2 cell fate determinants and a loss of asymmetry during the first division (Rappleye et al., 2003). In addition, PPK-1, a PI(4)P5 kinase, was found to accumulate at the posterior side of the one-cell embryo, where it would be responsible for an asymmetric generation of phosphatidylinositol-4,5-bisphosphate (PIP2), which in turn would lead to the recruitment at the posterior of LIN-5 and GPR-1/2, two factors required for asymmetric spindle positioning, in an unknown manner (Panbianco et al., 2008). The asymmetric distribution of plasma membrane PIP2 along the antero-posterior axis was confirmed recently and found to be dependent upon PAR determinants and F-actin (Scholze et al., 2018). Interestingly, PIP2 reciprocally regulates F-actin enrichment at one pole, which also appears to be required for proper polarity establishment and spindle positioning (Scholze et al., 2018).

The signaling activities of phospholipids are often mediated by phospholipases (Dennis, 2015). The seam cells in *C. elegans* have features of epithelial adult stem cells. In these cells, the catalytic activity of intracellular phospholipase A1 (iPLA1) is required for spindle orientation and asymmetry of cell fate specification during asymmetric divisions (Kanamori et al., 2008). This would be mediated, at least in part, by controlling the subcellular localization of β-catenin, which is involved in the establishment of polarity, through regulation of endosome-to-Golgi retrograde trafficking (Kanamori et al., 2008). *C. elegans* mutants for iPLA1 exhibit altered FA composition of PI, similar to mutants for 1-acylglycerol-3-phosphate O-acyltransferase (AGPAT) enzymes (Imae et al., 2010). As these AGPAT mutants present the same defects in seam cell asymmetric division and trafficking of β-catenin, this suggests a model in which altered FA composition of PI would cause abnormal localization of PIs in the membrane bilayer, leading to the mis-sorting of cortical proteins, such as β-catenin, by retrograde membrane trafficking (Imae et al., 2010).

In *Drosophila*, larval neuroblasts act as NSCs to generate all of the neurons and glia in adult flies. Recent data have shown that the PI regulating enzymes Vibrator and PI4 kinase IIIa (PI4KIIIa) are required for the asymmetric distribution of cell fate determinants in neuroblasts (Koe et al., 2018). Mutations that affect the PI binding and PI lipid transfer activities of Vibrator induce defects in neuroblast asymmetric division, leading to either the formation of ectopic neuroblasts or their loss through differentiation into neurons. The proposed model is that Vibrator stimulates PI4KIIIa to promote synthesis of phosphatidylinositol-4-phosphate (PI(4)P) at the plasma membrane, which, in turn, binds and anchors myosin to the neuroblast cortex, where it could recruit fate determinants.

The involvement of PIs in establishing polarity axis for asymmetric division of stem cells appears to be conserved in mammals. For instance, the asymmetric division of mouse epidermal stem cells depends on PI-dependent kinase 1 (PDK1). PDK1 is recruited to the apical side of the stem cell where phosphatidylinositol-3,4,5-trisphosphate (PIP3) is enriched, through the local activation of the PI3 kinase (PI3K) by interaction with E-cadherin at cell-cell contacts (Dainichi et al., 2016).

In addition to phospholipids, post-transcriptional lipid modifications, which constitute a prevalent mechanism for targeting proteins to membranes, may also participate in the establishment of cell polarity and asymmetric division. N-terminal N-myristoylation, the addition of a 14-C saturated FA, facilitates protein-plasma membrane interactions and is found on many proteins involved in asymmetric cell division (Benetka et al., 2008). Thus, myristoylation could possibly mediate the targeting of polarity and fate determinants to a given region of stem cell plasma membrane, although this remains to be investigated in more detail.

The above examples involve specific lipid species that directly maintain polarity of stem cells through interaction with cytoskeletal and other proteins. However, there are also some intriguing cases in which lipid metabolic pathways were shown to influence symmetric versus asymmetric outcomes of stem cell divisions, but the precise mechanisms involved remain to be elucidated. As noted above, in HSCs, a decrease in FAO leads to an increase of symmetric divisions giving rise to differentiating cells, which ultimately leads to exhaustion of the stem cell pool (Ito et al., 2012). Remarkably, deficiencies in FAO also increase the rate of mouse NSC symmetric differentiating divisions, at the expense of asymmetric divisions that normally maintain the pool of stem cells (Xie et al., 2016).

### Lipids and Interactions With the Niche

Adult stem cells reside in a specialized microenvironment, known as the niche, that provides signals essential to maintain “stemness” and survival (Voog and Jones, 2010; Scadden, 2014). Lipids could affect the interactions between stem cells and supporting cells within the niche in a variety of ways. Perhaps one of the most intuitive mechanisms would involve secretion of signaling lipids by niche cells; however, to our knowledge, there is only little evidence for such a mechanism. Osteoblasts are present in bone marrow and can participate in the regulation of HSCs by producing prostaglandin E2 (reviewed in Domingues et al., 2017). This lipid of the eicosanoid family binds to specific receptors at the surface of HSCs (Hoggatt et al., 2009) that mediate activation of the Wnt signaling pathway, which is important for HSC specification during development (Goessling et al., 2009) and for expansion of adult HSCs (Reya et al., 2003). In addition, HSCs also express receptors for the signaling lipid sphingosine-1-phosphate (S1P) that regulate localization within the bone marrow niche (Ogle et al., 2017).

Lipids can also influence niche-stem cell communication through regulation of signaling in niche cells. In *Drosophila*, subsets of niche cells in the ovary sense dietary cholesterol, which triggers the release and secretion of the signaling ligand Hedgehog to activate follicle stem cell (FSC) proliferation (Hartman et al., 2013). Interestingly, two studies in NSCs showed that LDs accumulating in adjacent cells affect NSC behavior in different ways. In a mouse model of Alzheimer’s disease, an accumulation of LDs was observed specifically in ependymal cells, the main support cells of the forebrain NCS niche, which correlated with a concomitant loss of NSCs (Hamilton et al., 2015). Through imaging MS-lipidomics in intact tissue sections, the authors determined that these LDs were selectively enriched in TAG containing oleic acid (18:1). Both *in vitro* and *in vivo*, an increase in oleic acid concentration negatively affects proliferation of NSCs via a mechanism that remains to be elucidated in detail but appears to involve activation of Akt signaling in NSCs. LDs were also observed to accumulate specifically in glial cells of the *Drosophila* larval central nervous system, which serve as niche cells for neuroblasts (Bailey et al., 2015). The number of LDs in glial cells increases under hypoxic conditions and in the presence of high levels of reactive oxygen species (ROS) (Bailey et al., 2015; Liu et al., 2015). Both FA synthesis and dietary FAs contribute to the formation of these LDs (Bailey et al., 2015; Liu et al., 2015). It was further shown that in response to high ROS levels, lipid biosynthesis increased in neurons, leading to increased transport of lipids from neurons to glia, where they are stored as LDs (Liu et al., 2017). Strikingly, glia-specific disruption of LD synthesis negatively affected neuroblast divisions during hypoxia, but not during normoxia (Bailey et al., 2015). Therefore, in this stem cell niche, LDs in adjacent glial cells are required to support stem cell divisions under adverse conditions, although the underlying mechanisms are still to be identified.

In other models, lipids and associated metabolic pathways have been shown to be required specifically for maintenance of niche cells and, in this way, affect stem cell maintenance in a non-autonomous manner. For example, secreted phospholipase A2, which produces lipid mediators such as lysophosphatidic acid and arachidonic acid, is specifically required for the differentiation and maturation of Paneth cells, which constitute a niche critical for ISC maintenance (Schewe et al., 2016). It is interesting to note that upon a HFD, or treatment with FAs or PPARδ agonists, ISCs show an increased capacity to form organoids *in vitro*, even in the absence of Paneth cells (Beyaz et al., 2016). One of the explanations proposed by the authors of this study is that activation of PPARδ upon excess FAs could induce the expression, in ISCs, of growth factors that are normally secreted and provided by the niche.

### Lipid Rafts and Signaling

Lipid rafts are dynamic membrane microdomains that are enriched in cholesterol and sphingolipids and act as platforms for signal transduction and membrane trafficking (Simons and Ehehalt, 2002). As differences in acyl chain composition of membrane phospholipids mediate their inclusion or exclusion from lipid rafts, this could be a mechanism to explain how different forms of phospholipids may have very specific effects on signaling (Imae et al., 2010). Therefore, lipid rafts dynamics are likely to contribute to the regulation of signaling pathways that are required for stem cell functions.

For example, lipid raft clustering is crucial for the regulation of HSC quiescence (Yamazaki et al., 2006). Re-entry of quiescent HSCs into the cell cycle (“activation”) requires the clustering of lipid rafts, which is mediated by cytokines, and inhibition of this process is sufficient to induce HSC quiescence. In this context, data suggest that lipid rafts regulate the PI3K–Akt–FOXO pathway in HSCs (Yamazaki et al., 2006). As most HSCs are quiescent in the bone marrow niche, lipid raft reorganization must be strictly regulated, likely by specific niche signals.

Also in the hematopoietic system, Hermetet et al. (2019) recently observed that lipid rafts are enriched in mouse HSCs, relative to more mature progenitor cells. Interestingly, a HFD leads to clustering and enlargement of lipid rafts, which affects the distribution of the TGFβ receptor within the lipid rafts. In this case, the clustering of the lipid rafts results in a decrease in TGFβ signaling, as it becomes concentrated in a smaller percentage of the membrane. As TGFβ signaling is essential for maintaining HSCs in a quiescent state, HFD leads to abnormal re-entry of HSCs into the cell cycle and ultimately causes their exhaustion (Hermetet et al., 2019).

Finally, a recent study reported that lipid rafts are present between niche cells and NSCs and suggested that the integrity of these lipid rafts was required for efficient niche-stem cell communication and NSC differentiation into neurons (Deng et al., 2019).

### Lipids and Regulation of the Transcriptome and Epigenome

At the molecular level, cell identity is controlled by a specific gene expression program, such that the transcriptome can be used to track cell fate decisions (Efroni et al., 2015). Activation and repression of gene expression are largely controlled by transcription factors and their interaction with the underlying epigenetic landscape. Stem cells express genes associated with self-renewal and potency (pluri-, multi-, uni-) and maintain a certain degree of plasticity in their epigenome to allow for differentiation (Bernstein et al., 2006; Voigt et al., 2013). In recent years, cellular metabolism has emerged as an essential regulator of the transcriptome and epigenome (Lu and Thompson, 2012; Etchegaray and Mostoslavsky, 2016), as specific metabolites can act as substrates or co-factors for epigenetic modifiers. In stem cells, the inter-relationship between metabolism and epigenetics plays important roles in the maintenance of potency and differentiation (Harvey et al., 2016; Atlasi and Stunnenberg, 2017; Mathieu and Ruohola-Baker, 2017; Fawal and Davy, 2018). Consequently, lipids and lipid metabolic pathways have the potential to regulate stem cell behavior by affecting the expression of stem cell-specific genes, through either a direct effect on transcription or modulation of epigenetic marks.

There are different mechanisms through which lipids can regulate gene expression (Zhdanov et al., 2016; Fernandes et al., 2018). First, *in vitro* experiments suggested that some lipid species, such as oleic acid, may directly bind to DNA (Zhdanov et al., 2002), which could have significant impacts on gene expression but is not currently supported by data obtained under physiological conditions (Fernandes et al., 2018). There is also some evidence supporting an effect of specific lipid species on DNA polymerase activity (Novello et al., 1975; Kamath-Loeb et al., 2014). Interestingly, although the mechanism remains unclear, changes in palmitate concentration *in vitro* appear to regulate the expression of neuronal genes and increased intracellular palmitate results in increased differentiation of neural precursor cells into neurons (Ardah et al., 2018). However, lipids primarily regulate gene expression through transcription factors, such as nuclear receptors, for which ligands include FAs and cholesterol metabolites. Nuclear receptors are activated by their ligands, bind to promoter/enhancer region of target genes and recruit co-regulators to activate or repress transcription. One of the main families of lipid-activated nuclear receptors is the PPARs. PPAR-regulated genes are mostly genes involved in lipid and carbohydrate metabolism (Barrera et al., 2008). Interestingly, signaling through receptors of the PPAR family was shown to be required for the maintenance of several types of adult stem cells. In the nervous system, this could involve direct regulation of genes associated with “stemness.” Specifically, PPARs are expressed in NSPCs of the SVZ and are required *in vitro* for proliferation and maintenance of the undifferentiated state (Bernal et al., 2015). Indeed, in NSPCs, PPARs appear to regulate the expression of the self-renewal gene *Sox2*, likely at the transcriptional level, given that the *Sox2* locus contains PPAR responsive elements (Bernal et al., 2015). This suggests that lipids and lipid-bound nuclear receptors may directly regulate the expression of stem cell specific genes. It would be interesting to assess whether PPAR responsive elements are enriched in the promoter regions of genes involved in the maintenance of self-renewal and multipotency in other adult stem cell models as well.

Similarly, the cholesterol-regulated transcription factor Sterol Regulatory Elements Binding Protein 2 (SREBP-2), which regulates the expression of cholesterol biosynthetic enzymes, was recently found to bind to the promoter regions of *notch1b* and other Notch target genes in *Danio rerio* (zebrafish) (Gu et al., 2019). In this study, transcriptional activation of the Notch pathway by SREBP-2 in conditions that promote hypercholesterolemia was shown to be essential for the mobilization of HSCs. Furthermore, SREBP-1, which regulates the expression of lipogenic genes, is expressed in human muscle cells in an insulin and growth factors dependent manner (Boonsong et al., 2007), despite a low lipogenesis rate in this tissue. Transcriptomics analyses revealed that SREBP-1 regulates the differentiation of satellite stem cells into myotubes by regulating the expression of two transcriptional repressors whose target genes include multiple myogenic genes (Lecomte et al., 2010). Therefore, transcription factors whose activity is modulated by lipids and/or lipid metabolism can contribute to the regulation of stem cell function via the control of target genes involved in establishing or maintaining specific cell identities.

Another crucial mechanism through which lipids can regulate gene expression is by mediating changes in the epigenome. One substrate used for histone acetylation is acetyl-CoA. While acetyl-CoA can be generated by various metabolic pathways, including glycolysis, amino-acid breakdown and FAO, a combination of proteomics and isotope tracing demonstrated that lipid metabolism is a major source of acetyl-CoA used for histone acetylation (McDonnell et al., 2016). Histone H3 acetylation correlates with “open chromatin” and was shown to be important for the maintenance of pluripotency and self-renewal of PSCs (Azuara et al., 2006; reviewed in Gaspar-Maia et al., 2011). In a recent study, the activation of lipid biogenesis pathways was identified as a conserved signature of naïve pluripotency in cultured hPSCs, mPSCs and in the human pre-implantation epiblast (Cornacchia et al., 2019). The maintenance in a naïve pluripotent state by lipid anabolism correlated with higher acetyl-CoA levels and increased histone acetylation, when compared to levels found in hPSCs in a primed pluripotent state, which do not show active lipid biogenesis (Cornacchia et al., 2019). Interestingly, however, examples of regulation of histone acetylation by lipids in adult stem cells were rare. As previously mentioned, activation of quiescent satellite cells is accompanied by a decrease in FAO, which leads to a reduction in intracellular NAD+ levels. This causes a decrease in SIRT1 activity and a subsequent increase in H4K16 acetylation, promoting the expression of muscle differentiation genes (Ryall et al., 2015). In addition, *in vitro* treatment of NSCs with palmitate resulted in a global increase in histone H3 acetylation, with specific enrichment at the promoter regions of some upregulated genes (Ardah et al., 2018). Notably, acetyl-CoA can also be reduced to produce the ketone body beta-hydroxybutyrate, which is an endogenous inhibitor of histone deacetylases (Shimazu et al., 2013). In NSCs, pharmacological inhibition of histone deacetylation promotes neuronal differentiation, while decreasing astrocyte differentiation *in vitro* (Fawal and Davy, 2018).

Although there is currently no direct evidence that lipids may bind to DNA to regulate gene expression, it is conceivable that lipids may bind to chromatin. For instance, cholesterol appears able to bind chromatin fibers, either directly or through proteins, and this interaction would promote the compaction of chromatin fibers, a state generally associated with transcriptional repression (Silva et al., 2017). A structural characterization, at the atomic level, of the lipids binding mode to nucleosomes would provide more insights into this issue (Fernandes et al., 2018).

Finally, LDs may also have the potential to affect gene expression via sequestration of proteins such as histones (Li Z. et al., 2012; Welte and Gould, 2017), which could ultimately cause changes in chromatin composition and underlie variations in gene expression. However, there is little evidence of a variation in LD accumulation during differentiation of adult stem cells that could be implicated in changes in gene expression. In *Drosophila* male GSCs, an abnormal increase of LDs resulting from perturbation of mitochondrial function appears to correlate with precocious differentiation (Sênos Demarco et al., 2019). A more detailed analysis and profile of LDs and their contents in stem cells and differentiating progeny could provide additional insights into a potential role of LDs in regulating stem cell function under homeostatic conditions.

## Dysregulation of Lipids and Effects on Stem Cells and Tissue Homeostasis

Diet, aging or disease, including cancer, can cause changes in lipid homeostasis. Accordingly, changes in the lipidome can be used to help in the diagnosis of a disease and/or provide potential therapeutic targets. In this section, we discuss how changes in the lipidome may affect endogenous stem cells. A better understanding of this relationship can provide insight into the pathophysiology of lipid imbalance disorders and improve therapeutic approaches on one hand and, on the other hand, could allow to potentially use lipid metabolites as therapeutic tools in order to modulate stem cell behavior in patients.

### Diet

Many studies have investigated how diet impacts the behavior of adult stem cells (Mana et al., 2017). HFD models in animals have been used to better understand the interplay between Western diets, which contain an excess of saturated fats, and the development of obesity and metabolic disorders (Wang and Liao, 2012).

Long term HFD (over 6 months) in mice triggers many of the metabolic phenotypes associated with obesity. A seminal study demonstrated that long term HFD reversibly increases the number and self-renewal capacity of mouse ISCs, at the expense of differentiation, in a cell-autonomous manner (Beyaz et al., 2016). Mechanistically, HFD leads to sustained activity of the PPARδ pathway and increased activity of the Wnt-β catenin pathway, which is required for stem cell maintenance. Treatment of gut organoids with FAs present in the HFD, *in vitro*, was sufficient to increase ISC number and self-renewal capacity, indicating that the effect of HFD on stem cells is unlikely to be an indirect consequence of obesity. As stem cells are considered to be more prone to acquire oncogenic mutations, this increase in “stemness” is proposed to be a mechanism contributing to the increase of tumor incidence observed upon HFD (Beyaz et al., 2016). Moreover, it was recently shown that HFD alters bile acids, which also contribute to drive malignant transformation of ISCs with dysregulated Wnt signaling (Fu et al., 2019). In *Drosophila*, increased dietary cholesterol influences the differentiation of ISCs by modulating Notch signaling, which leads to an increase of secretory entero-endocrine cells in the posterior midgut (Obniski et al., 2018).

In the hematopoietic system, it was suggested that long-term HFD (approximately 6 months) also increases the number and function of HSCs and skews differentiation toward the myeloid lineage, at the expense of the lymphoid lineage (Singer et al., 2014), contributing to inflammation and metabolic disease. In contrast, HFD administered for a short time (usually 4–6 weeks) appeared to cause a decrease in long-term repopulating HSCs, coupled with an increase in HSC proliferation and myeloid differentiation potential (Adler et al., 2014; Luo et al., 2015; Van Den Berg et al., 2016; Hermetet et al., 2019). However, the proposed underlying mechanisms differ among studies. Hermetet et al. (2019) observed a cell-intrinsic effect of HFD on HSCs: HFD promoted the clustering of lipid rafts at the surface of HSCs, which negatively affected TGF-β signaling and induced exit from quiescence and re-entry into the cell cycle, leading ultimately to exhaustion of the HSC pool. On the other hand, data from Luo et al. (2015) suggest that the effect of short-term HFD on HSCs is non-autonomous resulting from PPARγ-dependent changes in the bone marrow niche. Indeed, both short and long-term HFDs cause MSCs to differentiate primarily into adipocytes (Luo et al., 2015; Ambrosi et al., 2017; Tencerova et al., 2018). If this were to occur at the expense of osteoblasts, this could explain the increased risk of bone fracture in obese patients (Tencerova et al., 2018). Interestingly, contribution of the gut microbiome has also been proposed (Luo et al., 2015). These mechanisms are not necessarily incompatible; yet, it is unclear why long and short-term HFD would cause opposite effects on HSC number.

The increased consumption of saturated fats also contributes to neurodegenerative diseases, long-term memory loss and cognitive impairment (Park H. R. et al., 2010), which could be due, in part, to an effect on NSCs. For instance, the proliferation of NSPCs in the SVZ of the hippocampus is impaired upon HFD, without affecting neuronal and glial differentiation (Park H. R. et al., 2010). The decrease in proliferation appeared to involve increased lipid peroxidation and decreased secretion of a brain-derived growth factor. It was recently shown that in the SVZ, HFD decreases the proportion of NSPCs, at least in part, through the abnormal accumulation of senescent cells in the niche (Ogrodnik et al., 2019). Although it is not clear how these lipid-containing senescent cells alter the maintenance of stem cells, the systemic administration of senolytic drugs that eliminate senescent cells rescues, at least partially, the loss of NSPCs. When fed a HFD, mouse hypothalamic NSCs are also depleted, through ectopic activation of the IKKβ/NF-κB pathway (Li J. et al., 2012). In addition, differentiation of these multipotent cells into anorexigenic neurons expressing proopiomelanocortin (POMC neurons) is impaired and, because these neurons control satiety and insulin resistance, their loss upon HFD contributes to the development of obesity and pre-diabetes (Li J. et al., 2012).

Although the effects of HFD on adult stem cells have been investigated primarily in the gut, hematopoietic system and brain, there are examples of HFD-induced obesity also affecting other types of adult stem cells. For instance, HFD-induced obesity affects muscle regeneration in mice by inhibiting AMPK activity and preventing satellite cell activation (Fu et al., 2016). Given that AMPK can be activated by drugs, if this effect is conserved in humans, this finding raises the possibility of enhancing muscle regeneration in obese patients. However, it is unclear whether or not changes in lipid homeostasis are directly involved in this mechanism. In addition, although an increase in proliferation of stem cells in the lung was observed in response to short-term HFD, it was suggested that this may occur via an indirect mechanism. Indeed, supplementation of a regular diet with a lipid mix or individual lipids had no obvious effects (Hegab et al., 2018). Finally, epidermal stem cells undergo transcriptional changes in mice fed a HFD (Solanas et al., 2017; Lu Y. et al., 2019). Among the differentially expressed genes, genes regulating the extracellular matrix and the PI3K pathway may contribute to changes in niche signaling and altered stem cell function (Lu Y. et al., 2019). Yet, the detailed impacts of HFD on skin homeostasis remain to be investigated.

Overall, these studies suggest that short and long term HFD can impact the behavior of adult stem cells in a range of tissues; however, the mechanisms appear to differ. It will be interesting to determine whether the differences are associated with distinct changes in lipid homeostasis occurring in response to HFD and whether changes in stem cell behavior are a direct consequence of changes in lipid homeostasis upon HFD. In some cases, as for ISCs, treatment with FAs *in vitro* reproduced the effects of HFD (Beyaz et al., 2016), whereas is other cases, such as lung stem cells, this was not the case (Hegab et al., 2018). Importantly, in all cases in which it was assessed, the effects of HFD on stem cells appear to be reversible and could, therefore, potentially be corrected simply by changes in diet. Interestingly, while HFD seems to impair neurogenesis (Park H. R. et al., 2010; Li J. et al., 2012; Ogrodnik et al., 2019), specific types of FAs, including omega-3 FAs appear to increase neurogenesis (Kang et al., 2014; Nascimento et al., 2016; Lo Van et al., 2019), suggesting that diet could be used to rescue neuronal loss in diseases and aging.

### Inborn Errors of Metabolism

“Inborn errors of metabolism” encompass a large number of heterogeneous genetic diseases caused by rare mutations that affect the function of individual proteins, generally enzymes, involved in metabolic reactions. Among the inborn errors of metabolism affecting lipid metabolism, deficiencies in FAO have been shown to affect adult stem cells. Mutations in TMLHE, an enzyme catalyzing the first step of carnitine biosynthesis, are relatively frequent among this class of diseases and are associated with developmental neuropsychiatric disorders, including increased risk of autism. To better understand how a mutation that systemically affects lipid metabolism is linked with autism, a group of researchers specifically inactivated TMLHE in the mouse embryonic neocortex and observed a loss of NSCs (Xie et al., 2016). Strikingly, exogenous carnitine rescued at least partially this reduction of NSCs, whereas it had no effect in control animals. Thus, carnitine supplementation could represent a potential therapeutic approach to minimize the developmental brain deficits associated with inborn deficiencies in FAO (Xie et al., 2016).

### Aging and Degenerative Diseases

One striking hallmark of aging is altered stem cell behavior and, in some cases, stem cell exhaustion (López-Otín et al., 2013). In addition, there is increasing evidence that age-related changes in metabolism contribute to the age-associated decline in stem cell function (Ren et al., 2017). Lipid homeostasis is one of the metabolic parameters that are affected by aging. In many organisms, including in humans, aging is associated with increased fat storage and an altered membrane lipid composition that tends to decrease membrane fluidity (Gonzalez-Covarrubias, 2013). Moreover, membrane sphingolipids and the ratio of their metabolites sphingosine 1-phosphate (S1P) and ceramides change with age (Montoliu et al., 2014). These two signaling lipids mediate opposite cellular effects, S1P promoting proliferation and cell survival and ceramides promoting apoptosis. Interestingly, higher ceramides levels are associated with shorter lifespan in worms, and a decrease in S1P is often seen in age-related diseases such as Alzheimer’s disease (Papsdorf and Brunet, 2018). There is also evidence that the degree of unsaturation of membrane phospholipids gets higher with age, increasing lipid peroxidation products that may lead to more cellular damage (Papsdorf and Brunet, 2018).

However, the mechanism(s) by which changes in lipid metabolism could contribute to age-related changes in stem cell behavior remains largely unexplored. As mentioned above, lipidomics analyses of human MSCs identified changes in lipid profiles that could underlie the age-associated changes in MSC differentiation (an increase of adipocytes at the expense of osteoblasts), which contribute to the increase in osteoporosis in older individuals (Kilpinen et al., 2013; Lu X. et al., 2019). In these studies, lipid profiles were compared at different times across *in vitro* passaging of MSCs, as a way to model aging-related variations. Although this approach identified changes in lipids, the findings should be verified *in vivo.* Importantly, when comparing MSCs isolated from young and old donors, Kilpinen et al. (2013) did not describe significant differences. In addition, changes in lipid profiles with age appear to differ among tissues (Papsdorf and Brunet, 2018) potentially having different effects on the resident stem cells.

Aging is a primary factor for the development and progression of neuro-degenerative diseases (Hou et al., 2019), and alterations in lipid metabolism that impact NSC function have been shown to play a role in this process. For instance, in Alzheimer’s disease patients, oleic acid-enriched LDs accumulate in the forebrain, which correlate with a decrease in proliferation of NSCs (Hamilton et al., 2015). In a mouse Alzheimer’s model, pharmacological inhibition of the oleic-acid producing enzyme, stearoyl-CoA desaturase, was able to rescue the proliferation of NSCs (Hamilton et al., 2015). Although more investigation is required, this may offer a promising therapeutic approach to prevent cognitive decline and improve stem cell-mediated brain repair in Alzheimer’s disease.

Altogether, a better understanding of the relationships between altered lipid homeostasis and stem cell function during aging will contribute to the identification of new therapeutic targets for age-associated pathologies that arise due to changes in stem cell behavior.

## Summary and Perspectives

An increasing number of studies have implicated lipid metabolism, as well as individual lipid species, in the regulation of adult stem cell behavior. Advances in lipidomics have opened up new approaches to identify stem cell-specific signatures and novel lipid biomarkers that can be used to identify and sort stem cells. Yet, there are only a few examples of lipidomics in adult stem cells, likely because isolating enough adult stem cells to perform lipidomics remains challenging. Studies in PSCs and MSCs suggest that stem cells may generally contain lipids with a higher degree of unsaturation, when compared to differentiating progeny. Once lipidomics becomes more sensitive and/or stem cell purification strategies are improved, it will be interesting to determine to what extent adult stem cells share a common lipidome. What is clear is that FAO and lipid biogenesis pathways appear to be common regulators of adult stem cell behavior. In various types of adult stem cells, FAO is essential for stem cell maintenance, while *de novo* lipid synthesis promotes stem cell proliferation and differentiation. However, the mechanisms through which these lipid metabolic pathways affect stem cell behavior differ. Lipid catabolism and anabolism modulate the availability of different lipid intermediates and secondary metabolites that can affect stem cells in numerous ways, including regulation of self-renewal and multipotency via changes in signaling and gene expression. As one example, higher FAO activity in stem cells could lead to an increase in acetyl-CoA that can serve as a substrate for histone acetylation and participate in the maintenance of stem cell identity. In addition, *de novo* lipid biogenesis can regulate the pool of signaling lipids, such as PIs, that are involved in cell polarity and asymmetric division. Additional lipidomics and metabolomics analyses on purified adult stem cells should provide further insights into the contribution of specific lipid species to these mechanisms. Such studies will provide a better understanding of how lipid imbalances can affect adult stem cells, thereby contributing to changes in tissue homeostasis and the pathophysiology of diseases.

## Author Contributions

All authors listed have made a substantial, direct and intellectual contribution to the work, and approved it for publication.

## Conflict of Interest

The authors declare that the research was conducted in the absence of any commercial or financial relationships that could be construed as a potential conflict of interest.

## Abbreviations

ACC: Acetyl-CoA carboxylase
AGPAT: 1 -acylglycerol-3-phosphate O-acyltransferase
AMPK: AMP activated kinase
Cer: Ceramide
CPT1: Carnitine palmitoyltransferase I
CSC: Cancer stem cell
DHCer: Dihydroceramides
EB: Embryoid body
ETC: Electron transport chain
FA: Fatty acid
FAO: Fatty acid oxidation
FASN: Fatty acid synthase
FSC: Follicle stem cell
GSC: Germline stem cell
hESC: Human embryonic stem cell
HFD: High fat diet
HSC: Hematopoietic stem cell
iPLA1: Intracellular phospholipase A1
iPSC: Induced pluripotent stem cell
ISC: Intestinal stem cell
LD: Lipid droplet
Lpcat3: Lysophosphatidylcholine acyltransferase 3
mESC: Mouse embryonic stem cell
MS: Mass spectrometry
MSC: Mesenchymal stem cell
NSC: Neural stem cell
NSPC: Neural stem/progenitor cell
PC: Phosphatidylcholine
PDK1: PI-dependent kinase 1
PE: Phosphatidylethanolamine
PI: Phosphoinositides
PI3K: PI3 kinase
PI4KIIIa: PI4 kinase IIIa
PIP2: Phosphatidylinositol-4,5-bisphosphate
PIP3: Phosphatidylinositol-3,4,5-trisphosphate
PPAR: Peroxisome proliferator-activated receptor
PPRE: Peroxisome proliferator hormone response element
PS: Phosphatidylserine
PSC: Pluripotent stem cell
PUFA: Polyunsaturated fatty acid
RXR: Retinoid X receptor
S1P: Sphingosine-1-phosphate
SREBP: Sterol regulatory elements binding protein
SVZ: Subventricular zone
TAG: Triacylglycerol
TCA: Tricarboxylic acid
TOR: Target of Rapamycin
UPLC: Ultra performance liquid chromatography.
